# Successful treatment of cutaneous sarcoidosis with deucravacitinib, a selective TYK2 inhibitor

**DOI:** 10.1016/j.jdcr.2025.10.058

**Published:** 2025-11-05

**Authors:** Henry O. Herrera, Carol Sanchez, Gabrielle Minor, Gregory J. Fulchiero

**Affiliations:** a Department of Dermatology, Case Western Reserve University School of Medicine, Cleveland, Ohio; b Department of Dermatology, Pennsylvania State Health Hershey Medical Center, Hershey, Pennsylvania; c Keystone Dermatology and Center for Skin Surgery, Altoona, Pennsylvania; d Departments of Internal Medicine and Specialized Surgery, UPMC Altoona Hospital, Altoona, Pennsylvania

**Keywords:** cutaneous sarcoidosis, deucravacitinib, immunomodulation, JAK-STAT pathway, sarcoidosis, TYK2 inhibitor

## Introduction

Sarcoidosis is a multisystem disease characterized by noncaseating granulomas. Although the lungs and intrathoracic lymph nodes are the most commonly involved sites, sarcoidosis can affect nearly any organ system.^1^ Sarcoidosis is believed to result from an aberrant immune response to an unknown antigen in genetically susceptible individuals. The immunopathogenesis of sarcoidosis is characterized by the activation of CD4+ T-helper cells, leading to the production of pro-inflammatory cytokines, including interferon-gamma (IFN-γ), tumor necrosis factor-alpha (TNF-α), and interleukin-6 (IL-6).^2^ The cytokines produced by these T-helper cells promote macrophage activation and granuloma formation.^1^ Given the critical role of these inflammatory pathways, targeted inhibition of cytokine signaling has been explored as a therapeutic strategy.

Treatment options for sarcoidosis are limited. Corticosteroids are first-line therapy, however long-term use is associated with significant adverse effects, prompting the use of steroid-sparing agents such as methotrexate, azathioprine, and TNF-alpha inhibitors. More recently, Janus kinase (JAK) inhibitors, including tofacitinib and ruxolitinib, have demonstrated efficacy in refractory sarcoidosis by targeting cytokine-driven inflammation via the JAK-STAT pathway.^3^

In this case report, we describe the first known instance of cutaneous sarcoidosis being successfully treated with deucravacitinib, a selective tyrosine kinase (TYK) 2 inhibitor.

## Case presentation

**Fig 1:**
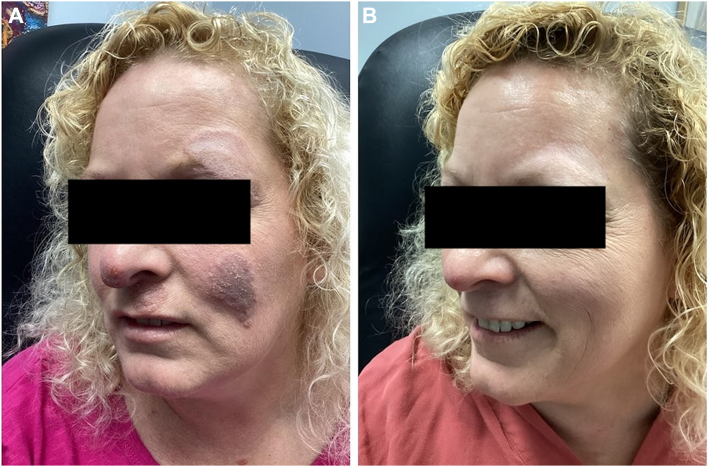
**A,** Clinical presentation of cutaneous sarcoidosis prior to treatment with deucravacitinib, showing a violaceous scaly plaque involving the left malar cheek and periorbital area. **B,** Significant clinical improvement after deucravacitinib therapy, with near-complete resolution of cutaneous lesions.

We present a 46-year-old female who presented with a 10-year history of facial rash and a remote history of deep vein thrombosis. Physical exam revealed indurated, annular pink to brown plaques primarily affecting the left malar cheek, upper lip, and left eyebrow (Fig 1, *A*). Initial biopsy demonstrated non-caseating granulomas and perifollicular lymphocytes and histiocytes. Special stains for acid-fast bacilli and fungi were negative. Findings favored granulomatous rosacea. Repeat biopsies after several failed therapies revealed similar histologic findings. Concomitantly, she exhibited shortness of breath and cough, which led to imaging that revealed multiple pulmonary nodules, along with mediastinal and hilar adenopathy with coarse central calcifications. The constellation of systemic symptoms, cutaneous manifestations, histologic findings, and lack of response led to a clinical diagnosis of sarcoidosis. Therapies were trialed for 4 to 6 months and included topical corticosteroids, doxycycline (100 mg BID), and hydroxychloroquine (200 mg BID) in 2019. In 2020, she was placed on dapsone (100 mg BID) and topical dapsone. In 2021, trials of methotrexate (10 mg qWK) were not effective in halting the progression of her disease. She experienced mild improvement with halobetasol propionate 0.01% topical lotion in 2022. After a thorough discussion of biologic options, deucravacitinib 6 mg daily was initiated. Within several months, she demonstrated marked clinical improvement, with near-total resolution of her cutaneous lesions, along with improvement in dyspnea and cough (Fig 1, *B*).

## Discussion

Deucravacitinib is an oral TYK2 inhibitor that binds to the regulatory domain of TYK2 and is FDA-approved for moderate-to-severe plaque psoriasis. Unlike JAK inhibitors, TYK2 inhibitors do not carry the same risk of venous thromboembolism or major adverse cardiovascular events, which informed our decision to pursue this therapy for a patient with a remote history of deep vein thrombosis.

To the best of our knowledge, this is the first reported case of sarcoidosis treated with a TYK2 inhibitor. Deucravacitinib is also being studied for systemic lupus erythematosus. In a Phase II randomized, double-blind, placebo-controlled trial, Morand et al demonstrated that deucravacitinib significantly improved lupus disease activity scores compared to placebo, with a favorable safety profile and reduced risk of broad immunosuppression.^4^ Other TYK2 inhibitors, such as lomedeucitinib and ESK-001, are currently in clinical development for plaque psoriasis.^5^

TYK2 plays a crucial role in signaling pathways mediated by IL-12, IL-23, and IFN-α/β. These cytokines are key drivers of Th1 and Th17 responses, which contribute to the pathogenesis of sarcoidosis.^6^ IL-12 production through TYK2 and JAK2 induces naïve CD4+ T cell differentiation into Th1 cells, leading to the production of IFN-γ. This cytokine further activates macrophages, enhancing granuloma formation. IL-23 signaling through TYK2 and JAK2 sustains the Th17 response, leading to IL-17 and IL-22 production, which contribute to chronic inflammation and granuloma maintenance.^6^ IFN-γ is a key driver of granuloma persistence in sarcoidosis. Elevated IFN-γ levels activate macrophages, inducing the secretion of TNF-α, IL-6, and IL-12, which amplify the inflammatory cascade.^7^ Deucravacitinib does not directly inhibit the production of IFN-γ but rather decreases levels of IL-12 through TYK2 inhibition. This reduces the activation of Th1, leading to less production of IFN-γ. Given the central role of these pathways in granuloma formation, inhibiting TYK2 may provide a more targeted approach to modulating immune responses in sarcoidosis compared to the inhibition of JAK inhibitors. JAK inhibitors block multiple JAK isoforms, affecting a wide range of cytokines, including IL-6, IL-12, and IFN-γ. This results in broad immunosuppression, which can be effective in treating sarcoidosis.^3^^,^^8^ Tofacitinib is a JAK1/3 inhibitor that has shown efficacy in refractory sarcoidosis. A patient with multiorgan involvement demonstrated complete resolution of cutaneous and internal granulomatous lesions after 6 months of therapy.^3^ In 1 small open-label trial, treatment with tofacitinib for 6 months showed improvement in cutaneous and internal organ disease. Total lesion glycolysis, a measure for active internal organ sarcoidosis, decreased by more than 50% in 5 patients on tofacitinib and 3 patients had near-total resolution.^7^ Ruxolitinib, a JAK1/2 inhibitor, has demonstrated efficacy in refractory sarcoidosis. A patient with an associated JAK2 mutation showed significant clinical and radiographic improvement following treatment.^9^

Deucravacitinib is proposed to have an improved safety profile compared to JAK inhibitors due to its selectivity for TYK2. There was no evidence of hematological or lipid abnormalities in the experimental group of the POETYK phase III trials. The most frequently reported adverse events were nasopharyngitis, upper respiratory illness, and COVID-19. Three patients developed venous thromboembolism, and 3 developed lymphoma, but neither was found to be treatment-related.^10^

As the therapeutic landscape for TYK2 inhibition expands, they may pave the way for further investigations into their role in immune-mediated diseases. The growing body of research on TYK2 inhibitors highlights their potential as a targeted immunomodulatory strategy, warranting clinical trials to assess their efficacy beyond plaque psoriasis. TYK2 inhibition may represent a promising new therapeutic avenue for sarcoidosis.

## Conflicts of interest

None disclosed.
